# Contrasting impacts of precipitation on Mediterranean birds and butterflies

**DOI:** 10.1038/s41598-019-42171-4

**Published:** 2019-04-05

**Authors:** Sergi Herrando, Nicolas Titeux, Lluís Brotons, Marc Anton, Andreu Ubach, Dani Villero, Enrique García-Barros, Miguel L. Munguira, Carlos Godinho, Constantí Stefanescu

**Affiliations:** 1 Catalan Ornithological Institute, Natural History Museum of Barcelona, Plaça Leonardo da Vinci 4–5, 08019 Barcelona, Catalonia Spain; 2 CREAF, 08193 Cerdanyola del Vallès, Spain; 3 German Centre for Integrative Biodiversity Research (iDiv), Halle-Jena-Leipzig, Deutscher Platz 5e, 04103 Leipzig, Germany; 4 Helmholtz Centre for Environmental Research (UFZ), Department of Community Ecology, Theodor-Lieser-Str. 4, 06120 Halle, Germany; 5 InForest Jru (CTFC-CREAF), Crta. Antiga St Llorenç de Morunys km 2, 25280 Solsona, Catalonia Spain; 6 CSIC, Cerdanyola del Vallès, Spain; 7 Museum of Natural Sciences of Granollers, Francesc Macià 51, 08402 Granollers, Catalonia Spain; 8 Universidad Autónoma de Madrid, Centro de Investigación en Biodiversidad y Cambio Global, Departamento de Biología, c/Darwin 2, 28049 Madrid, Spain; 9 ICAAM (Instituto de Ciências Agrárias e Ambientais Mediterrânicas) – LabOr (Laboratório de Ornitologia), Universidade de Évora. Pólo da Mitra, Apartado 94, 7002-774 Évora, Portugal

## Abstract

The climatic preferences of the species determine to a large extent their response to climate change. Temperature preferences have been shown to play a key role in driving trends in animal populations. However, the relative importance of temperature and precipitation preferences is still poorly understood, particularly in systems where ecological processes are strongly constrained by the amount and timing of rainfall. In this study, we estimated the role played by temperature and precipitation preferences in determining population trends for birds and butterflies in a Mediterranean area. Trends were derived from long-term biodiversity monitoring data and temperature and precipitation preferences were estimated from species distribution data at three different geographical scales. We show that population trends were first and foremost related to precipitation preferences both in birds and in butterflies. Temperature preferences had a weaker effect on population trends, and were significant only in birds. The effect of precipitation on population trends operated in opposite directions in the two groups of species: butterfly species from arid environments and bird species from humid habitats are decreasing most. Our results indicate that, although commonly neglected, water availability is likely an important driver of animal population change in the Mediterranean region, with highly contrasting impacts among taxonomical groups.

## Introduction

Evaluating which species will be most vulnerable to climate change is of utmost importance to guide anticipative management strategies for biodiversity conservation^1^. The link between the climatic requirements of the species and their observed population trends provides useful information for estimating their vulnerability to future climate change^2^. With long-term monitoring schemes covering large geographical areas, birds and butterflies are among the groups of animals that are most often used to carry out such analyses^3–9^. Some assessments focusing on the temperature requirements of the species have shown that populations of cold-dwelling species are declining faster than those of warm-dwelling species^3,6^. Yet, these studies largely ignore that part of the climatic preferences of the species related to their precipitation requirements^4^. In regions in which water is a limiting resource and where the precipitation regime is changing with increasingly frequent and intense drought events, rainfall requirements may play a role that is at least as important as temperature in determining population trends. Therefore, neglecting this component of the climatic requirements of the species in such assessments could lead to a biased estimation of their vulnerability to future climate change.

In this study, we used long-term monitoring data on birds and butterflies in Catalonia (NW Mediterranean Basin) to analyse whether observed population trends of the species in the last decades can be predicted from descriptors of their temperature and/or precipitation preferences. In the Mediterranean Basin, both temperature and water availability constitute a critical constraint for ecosystem functioning and their effects are likely to increase in the near future^10–12^. Temperature has indeed experienced an increase over recent decades and the overall amount of rainfall has been steadily decreasing, particularly during summer^13–15^. Interestingly, previous studies on Mediterranean birds and butterflies have not shown a clear link between the thermal preferences of the species and their recent population trends suggesting that other factors beyond temperature changes alone are behind these biological responses^3,16,17^. Hence, we examined if the observed trends of these species are explained by their rainfall requirements better than by their temperature requirements.

The climatic requirements of the species can be estimated at varying geographical scales and spatial resolutions, but there is no consensus on which scale is the most adequate when linking this information with local population trends. Hence, we used distribution data at three geographical scales (Europe, Iberian Peninsula and Catalonia) to calculate two indices reflecting the precipitation and temperature requirements of each individual bird or butterfly species^3^: the mean annual precipitation across the geographical range it occupies (Species Precipitation Index, SPI) and the mean annual temperature across its range (Species Temperature Index, STI). In order to evaluate the strength of the relation between the long-term population trends of the species and these two indices across spatial scales, we developed linear models at each scale and we used an information-theoretic model selection approach to identify the scale at which this link was the strongest.

As climate change may interact with other relevant drivers such as habitat modifications due to land use changes^18–20^, we controlled for the impact of the increase in forest cover across the landscape. Vegetation encroachment after the abandonment of traditional agricultural practices in the study area has been a major driver of biodiversity change during the last decades^21^. Therefore, we incorporated an additional index in the models reflecting the preference of the species along the gradient from open to forest habitats (Species Afforestation Index, SAFI)^21^. We evaluated the extent to which this index explained the observed population trends of the species in addition to and in interaction with the two indices reflecting climatic preferences.

## Results

**Figure 1:**
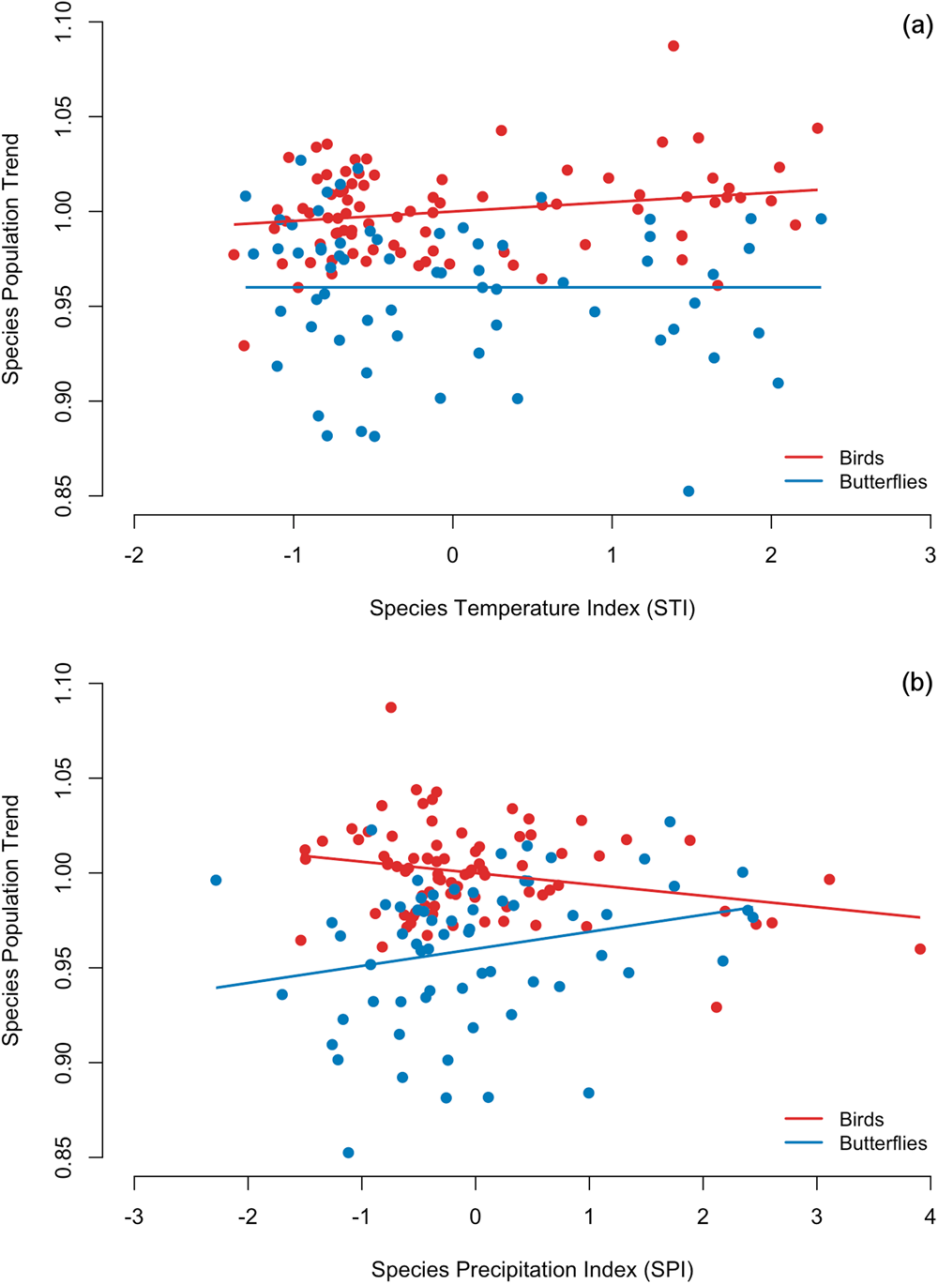
Population trends and climatic preferences. The graphs show the relationships between the population trends of the bird (red) or butterfly (blue) species in Catalonia and their climatic preferences: (**a**) Species Temperature Index at European level (STI) and (**b**) Species Precipitation Index at Iberian level (SPI). The relationships are estimated from an AICc-based multi-model inference procedure (Table 2).

We found that the index related to precipitation preferences of the species at Iberian scale (SPI[ibe]) was the strongest predictor of recent population trends for both birds and butterflies. In contrast, the effect of temperature preferences was only supported in the models developed for birds and this effect was better captured at the European scale (STI[eur]) (Tables 1 and 2; Fig. 1). For birds, a model without STI[eur] was within the set of supported models but this model was associated with a considerable decrease in log-likelihood, indicating that this parameter was less strong than SPI[ibe] but still informative^22^.

**Table 1:** Set of supported (∆AICc < 2) and best non-supported (∆AICc > 2, between brackets) candidate models for population trends of bird and butterfly species, with their relative fit (log-likelihood) and support (AICc weight and Sum of AICc weights) according to the model selection procedure.

Analyses	Supported and (best non-supported) models		K	Log-likelihood	∆AICc	AiCc weight	Sum AICc weights
	Main effects	Interaction effects					
Butterflies	SPI[ibe] + SAFI		4	123.2	0	0.269	0.269
	SPI[ibe]		3	121.7	0.816	0.179	0.449
	SPI[ibe] + SAFI	SPI[ibe] × SAFI	5	123.3	2.198	0.09	0.538
Birds	SPI[ibe] + STI[eur] + SAFI	STI[eur] × SAFI	6	201.9	0	0.206	0.206
	SPI[ibe] + STI[eur] + SAFI		5	200.7	0.178	0.188	0.394
	SPI[ibe] + SAFI		4	198.6	1.969	0.077	0.47
	SPI[ibe] + STI[eur] + SAFI	STI[eur] × SPI[ibe] + STI[eur] × SAFI	7	202.1	2.142	0.07	0.541

K: number of parameters estimated in the model.

Log-likelihood: relative measure of model fit.

∆AICc: difference in AICc between any candidate model and the best model associated with the smallest AICc.

AICc weight: weight of evidence that the candidate model is the best model.

Predictors: STI[eur] = Species Temperature Index at European scale, SPI[ibe] = Species Precipitation Index at Iberian scale, SAFI = Species Afforestation Index.

**Table 2:** Results of the AICc-based multi-model inference procedure examining the variations in butterfly and bird species population trends relative to their climatic and land use preferences (predictors).

Effects	Predictors	Butterflies					Birds				
		freq	w	p	coef	se	freq	w	p	coef	se
(Intercept)		1	1	1	0.96	0.005	1	1	1	1	0.003
**Main effects**											
	STI[eur]	0.722	0.358	0.643	0	0.002	0.722	0.86	0.04	0.005	0.003
	SPI[ibe]	0.722	0.809	0.047	0.009	0.004	0.722	0.894	0.03	−0.006	0.003
	SAFI	0.722	0.673	0.135	0.005	0.004	0.722	0.866	0.038	0.005	0.002
**Interaction effects**											
	STI[eur] × SPI[ibe]	0.278	0.062	0.451	0	0	0.278	0.191	0.096	0	0.001
	STI[eur] × SAFI	0.278	0.065	0.433	0	0	0.278	0.401	0.034	0.002	0.002
	SPI[ibe] × SAFI	0.278	0.135	0.162	0	0.001	0.278	0.192	0.101	0	0.001

Predictors: STI[eur] = Species Temperature Index at European scale, SPI[ibe] = Species Precipitation Index at Iberian scale, SAFI = Species Afforestation Index.

freq: frequency of the different predictors in the list of candidate models.

w: level of importance of the predictor for explaining the data (range: 0–1).

p: probability that by chance w is as high as the estimated value (based on 1000 permutations).

coef and se: estimated parameters and their unconditional standard errors.

Our results are consistent with the idea that recent trends in Mediterranean populations are explained to a large degree by species requirements related to precipitation and water availability patterns. Importantly, we found that the effect of precipitation requirements on population trends operated in opposite directions for these two groups. Butterfly species from dry environments (i.e., associated with low SPI values) and bird species from humid areas (i.e., associated with high SPI values) have undergone the most severe population declines during recent decades (Table 2; Fig. 1). In the case of birds, species from cold environments (i.e., associated with low STI values) are also declining more than those from warm areas (Table 2; Fig. 1).

SAFI was included in all supported models for birds (Table 1). For butterflies, the best model included SAFI and the supported model without SAFI did not substantially increase AICc but considerably reduced log-likelihood, which indicated that SAFI was also informative to explain population trends^22^ (Table 1). Butterfly and bird species from open areas (i.e., associated with low SAFI values) have declined more markedly than forest-dwelling species.

The interactive effects of SPI[ibe], STI[eur] and SAFI were not supported by the model selection procedure in the case of butterflies. For birds, the best model included the interaction STI[eur] × SAFI and its effect was supported in addition to the main effects (Tables 1 and 2). This interaction showed that birds from cold and open areas have declined more than those from cold and forest areas.

Similar results were obtained in additional analyses taking into account the mobility and the degree of taxonomic relatedness of the species (see Supplementary Information).

## Discussion

We showed that the descriptor capturing the precipitation preferences of the species was the strongest predictor of population trends both for Mediterranean bird and butterfly species. Our results also indicate that rainfall requirements have a different impact on these two groups of animals: butterfly species from arid environments are decreasing more severely than those of humid habitats, while the pattern is the opposite for birds. Beyond this dominant effect of precipitation requirements, we also found that birds from cold areas have undergone overall steeper declines than birds from warm areas in Catalonia, a pattern found previously at the continental level^3,8^. Our results also highlight the importance of exploring the simultaneous impact of climate change and habitat dynamics on biodiversity. In our study system, afforestation resulting from land abandonment has also clearly affected bird and butterfly population changes.

The main finding of this study is the identification of the key role played by precipitation requirements in recent bird and butterfly population trends. Further research is however needed to fully understand the different mechanisms through which water availability impacts on Mediterranean biodiversity, as this factor seems to limit different groups of species in contrasting ways. Butterflies are ectotherms with limited mobility during their immature stages^23^. Hence, they may be at great risk of exceeding physiological tolerance thresholds during extreme drought events^24^. In the study area, there has been a significant decrease in summer precipitation^15^ and an increase in consecutive dry days over the last few decades^25^. Mortality of early life stages (i.e. eggs or early larval instars) due to desiccation stress during drought events has been shown as a key determinant of butterfly vulnerability to climate change^26^. The strongest population decreases in butterflies from dry lowland areas might therefore be related – at least partly – to direct physiological limitations when facing water shortage. This possibility seems highly likely in view of a recent study showing declines of lowland populations but stability of mountain populations in a common butterfly species in the region, as a consequence of an important reduction in vegetation thermal buffering effects under summer drought at low elevation habitats^27^. In addition, drought-stressed host plants may also provide lower-quality food resources to the larvae with a carry-over effect on the reproductive performance during the adult stage of the butterflies^28^, which may then have an impact on the population growth rates of these species.

Direct physiological limitations linked to aridity are probably affecting birds less strongly because they are endotherm organisms^29^. However, variations in rainfall regime may have an important impact on bird populations^30^. With their high trophic level, birds potentially face the risks of phenological mismatches between the timing of their reproduction and optimal climate conditions for food provisioning to the nestlings during the breeding period^31^. These risks could be particularly important in ecosystems with narrow temporal windows for breeding, in which such mismatches may have significant impacts on the population dynamics^32^. In our study area, temporal windows for breeding are particularly short in humid and cold upland areas. Lower water availability during summer in these areas^13^ may have an effect on soil moisture and vegetation and this may impact invertebrate communities that constitute key prey items for many of the bird species during the breeding season. Our finding is consistent with the results of a recent study that has showed the decline of mountain bird populations across Europe^33^ and suggests that phenological mismatches related to decreases in the precipitation could partly explain why mountain birds have been showing the most severe declines during recent decades.

The scale at which temperature and precipitation requirements are estimated may affect the possibility of finding a link with species population trends in a geographically limited study area such as Catalonia. This scale issue definitely warrants further investigation because it is largely unclear whether it is more adequate to examine local population trends in the light of broad species requirements estimated across their whole range of distribution or at smaller and finer spatial scale. We found that precipitation requirements calculated at continental and local scales were not as good predictors of population trends as precipitation requirements estimated at the scale of the Iberian Peninsula. In contrast, temperature requirements had more predictive power when estimated at a continental scale, at least in the case of birds. These results suggest that the relevance of the spatial scale when linking population trends with climatic requirements may depend on the climatic dimension itself. Our study focuses on a Mediterranean area where water availability is a key constraint. This may explain that a regional scale is more suited to estimate precipitation requirements of the species and their link with local population changes than estimations carried out at larger scales. This would suggest that at least some dimensions of climatic requirements of the species estimated at very large scales may not always reflect the actual constraints on species biology and therefore limit our capacities to link these constraints with the observed population trends.

Our study highlights the need for examining precipitation regimes when exploring the link between biodiversity dynamics and climate change, especially in regions where water availability is a limiting factor due to recurring precipitation deficits such as the Mediterranean Basin^13^. It shows the importance of considering precipitation when trying to understand recent population trends and projecting them under future climate change scenarios because species might respond to shifts in the rainfall regime more than to increasing temperatures. Changes in precipitation have certainly a significant overall impact on biodiversity in some regions, and we also demonstrate here that this effect may greatly differ between groups of species depending on their ecology and life history traits. Hence, we encourage researchers to place a stronger emphasis on precipitation when examining the effects of climate change on biodiversity and to avoid drawing general conclusions derived from patterns observed in a single group of species.

## Methods

**Figure 2:**
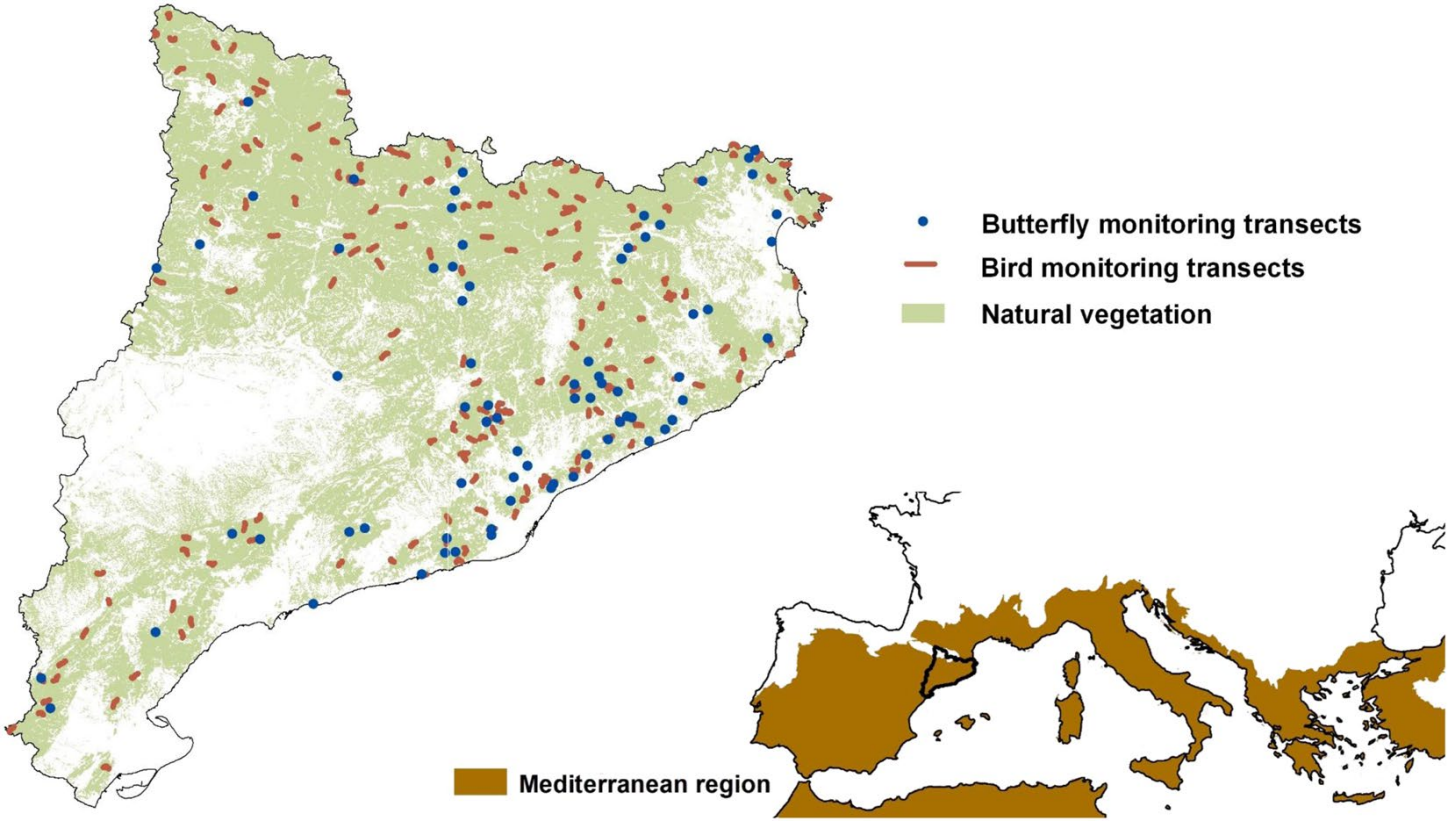
Location of monitoring transects and the study area. Bottom-right map: location of the study area (Catalonia) in Southern Europe along with the Mediterranean region according to Metzger *et al*.^34^. Upper-left map: location of selected monitoring transects from the Catalan Breeding Bird Survey (CBBS) and the Catalan Butterfly Monitoring Scheme (CBMS) within the areas mostly covered by natural (or semi-natural) vegetation in the study area. This selection of sites was done in order to minimise the effect of anthropogenic pressures not addressed in this study. See main text for details.

The study was carried out in Catalonia (NE Iberian Peninsula) (Fig. 2), an area in the NW of the Mediterranean Basin with a broad gradient of climatic conditions within the European context^34^.

The standard Breeding Bird Survey (BBS) and Butterfly Monitoring Scheme (BMS) projects represent the core of biodiversity monitoring in Europe, since these are the only groups for which harmonized monitoring data are available over large geographical areas^35^. In this study bird and butterfly population trends were estimated using long-term monitoring data from the Catalan versions of BBS (CBBS) and BMS (CBMS) initiated in 2002 and in 1994, respectively. In this study, we selected data for the period 2002–2016 in order to maximise the comparability of analyses and results between birds and butterflies. CBBS field methodology is based on linear transects of ca. 3,000 m that are surveyed twice a year during the breeding period (15 April–15 June)^36^. For each breeding bird species, the maximum count recorded during these two surveys is retained as the best estimation of its annual abundance in each transect. CBMS data collection is also based on linear transects and observers count the butterflies detected within a 5 × 5 m virtual area along the line of progression^37^. Transects vary in length, with a mean of ca. 1,700 m. Butterfly surveys are carried out during 30 consecutive weeks from March to September and the sum of the individuals recorded during the surveys for a species (including statistically estimated values for missing weeks) is retained as the best estimate of its annual abundance in each transect.

We selected only CBBS and CBMS monitoring sites located in natural or semi-natural areas (i.e. grassland, shrubland and forest) to minimise the potential effect of human activities not addressed in this study on species living in highly anthropogenic habitats such as urban or agricultural areas. Only transects crossing at least 75% of natural or semi-natural habitats were included in this study (Fig. 2). In total, we considered 174 CBBS transects for birds and 74 CBMS transects for butterflies.

Species population trends were calculated using log-linear Poisson regression models implemented in the TRIM software^38,39^. In order to reduce the uncertainty in the analyses, our dataset did not include species whose trends could not be estimated, species occurring in a low number of sampled sites (<30 transects) or species with the most inaccurate population trends (5% of species with highest standard error around the trend estimates). In total, 83 bird species (37% of the set of native species breeding in Catalonia) and 64 butterfly species (32% of the set of native species in Catalonia) were included in the final analyses. Their average population trends were 0.999 (SD = 0.024) for birds and 0.960 (SD = 0.037) for butterflies, with a value of 1 indicating a stable abundance along the time series. The species retained for the analysis were considered as those associated with an acceptable level of uncertainty around their population trend estimates. We decided to assign the same weight to each of these species in the linear models relating trends with climatic preferences and not to weight the contribution of each species based on the standard errors of their trends. We run the analyses in this manner because these errors were smaller in species that are distributed across the entire study area and therefore present in a higher number of sampled sites. A weighting procedure would therefore give more importance in the analysis to widespread and generalist species along the gradient of climatic conditions, with a relative neglect of geographically and climatically more restricted species. We considered that such a weighted analysis would not be ecologically meaningful because the results would be largely driven by species that are not representative of the entire set of species with respect to their response to changing climate conditions.

Species Temperature Index (STI) and Species Precipitation Index (SPI) reflect the average annual temperature and precipitation experienced by species over their ranges and were calculated following Devictor *et al*.^3^. STI and SPI values were estimated using a global dataset on climatic conditions (period 1970–2000)^40^ and species distribution data at three different scales and resolutions. First, we estimated these climatic preferences for the whole of Europe (STI[eur] and SPI[eur]) using the species occurrences at 50-km resolution (period 1981–2000)^41,42^. Second, we did the same using the species occurrences at 10-km resolution in the Iberian Peninsula (period 1998–2005)^43–45^ (STI[ibe] and SPI[ibe]). Third, we used species occurrences within CBBS and CBMS monitoring sites in Catalonia at 1-km resolution (period 2002–2016) (STI[cat] and SPI[cat]).

In order to account for land use changes that could potentially impact on bird and butterfly population trends in the natural and semi-natural habitats of the study area^46^, we calculated for each species a measure of its preference along a gradient from open to forest habitats (Species Afforestation Index, SAFI). With this index, we controlled for the potential impacts of increasing forest cover in the region over the last few decades due to vegetation encroachment after land abandonment (see details in Herrando *et al*.^21^).

We used linear models to predict variations in bird and butterfly population trends from the indices reflecting the climatic (STI and SPI) and land use (SAFI) preferences of the species. We carried out these analyses for birds and butterflies separately. STI, SPI and SAFI predictors were standardised before the analyses (mean = 0 and SD = 1 at each spatial scale). We used an information-theoretic model selection approach^47^ based on the Akaike Information Criterion corrected for small sample sizes (AICc) to evaluate the strength of evidence for the relative influence of the predictors. The climatic predictors (STI and SPI) with the strongest predictive power were included in the analysis based on a hierarchical approach. For STI, we built models predicting variation in population trends from STI[eur], STI[ibe] and STI[cat] only and we retained the predictor producing the best fit (highest log-likelihood). The same procedure was applied to SPI. Once the most appropriate scale was identified for STI and SPI, we developed the full models including STI, SPI, SAFI and their interactions. All possible combinations of predictors were then derived from the full models to produce a set of candidate models. Interactions were only incorporated in a candidate model when both main effects were also included. The differences in AICc (∆AICc) were used to rank the candidate models relative to the best approximating model associated with the smallest AICc. A ∆AICc value < 2 was used as a threshold for a model to be considered as receiving support. The relative support for the candidate models was obtained by scaling them according to their AICc weight. We estimated the relative importance of a predictor (w) by summing the AICc weights across all candidate models in which the predictor occurred. We carried out permutation tests (number of permutations: n = 1000) to estimate the probability that the AICc weight of each predictor would be as high as the observed value by chance only^47^. We also examined the differences between each supported model and the best model in terms of number of parameters (K) and model fit (as measured by the log-likelihood value). If models were supported (∆AICc < 2) because they only included one additional parameter without improving model fit (similar log-likelihood values), the model was not regarded as competitive and the predictor linked to this additional parameter was considered as uninformative^22,47^. We used a model-averaging procedure to estimate the parameters (coef) and unconditional standard errors (se) for each predictor.

## Supplementary information

Supplementry Information

## Data Availability

The values of STI, SPI (at the three spatial scales) and SAFI for each species are available together with information on their population trends in the open DRYAD data repository linked to this manuscript. 10.5061/dryad.ch8dd57.
