# Supplementary material for: Contrasting impacts of precipitation on Mediterranean birds and butterflies
Source: Sci Rep. 2019 Apr 5;9:5680. doi: 10.1038/s41598-019-42171-4 (PMC6450943; doi:10.1038/s41598-019-42171-4)
Supplement: Supplementary file 1 — Supplementry Information [file 41598_2019_42171_MOESM1_ESM.docx]

**Contrasting impacts of precipitation on Mediterranean birds and butterflies. Supplementary Information.**

**Controlling for the potential influence of additional factors on population trends in birds and butterflies**

Sergi Herrando, Nicolas Titeux, Lluís Brotons, Marc Anton, Andreu Ubach, Dani Villero, Enrique García-Barros, Miguel L. Munguira, Carlos Godinho, Constantí Stefanescu

The effect of climate and land use changes on local population trends may be influenced by the amount of time the organisms spend in the study area during their lifespan. Mobile species could be potentially less affected by local drivers than species spending a substantial part of their life cycle within the study area. To control for that we classified species as mobile or not (Mobility: Yes/No) depending if they move beyond the study area or not during at least part of their life cycle. We also controlled for the degree of relatedness among species in order to determine if there is any taxonomic underpinning for the observed patterns. To do so, we included an additional categorical variable (Taxon: Family level for butterflies and Order level for birds, except for the large Order Passeriformes which was split between Infraorder levels). We included these two categorical variables (Mobility and Taxon) along with the predictors used in the modelling procedure described in the main text. Because of the very low number of species for some taxonomic levels, it was not possible to include their interactions with the other predictors.

With these additional analyses, the effects of STI[eur], SPI[ibe] and SAFI on species population trends were essentially the same as those estimated from the models presented in the main text (see Supplementary Tables S1 & S2 online). When controlling for the effect of Mobility and Taxon in the models, SPI[ibe] remained as the strongest predictor of recent population trends both for birds (coef. = -0.006, SE = 0.003) and butterflies (coef. = 0.012, SE = 0.006). STI[eur] was also an important predictor for birds (coef. = -0.005, SE = 0.003) but not for butterflies. SAFI was also informative for the prediction of population trends in birds (coef. = 0.006, SE = 0.003), but not in butterflies. The interactive effects STI[eur]×SAFI was supported for birds only.

For butterflies we found an effect of mobility on population trends (coef. = 0.025, SE = 0.009): mobile species tended to experience lesser declines than the ones completing their entire life cycle in the study area, maybe because mobile species are able to track more easily those areas that are less affected by drought. The effect of the taxonomic level was not supported for butterflies. In birds, population trends varied considerably among the taxonomical levels analysed, probably because some groups of birds are more capable to compensate the adverse effects of climate and land use change in the study area. For instance, corvides (coef. = -0.005, SE = 0.006) have more positive trends than passerides (coef. = -0.009, SE = 0.007), maybe as a result of their higher behavioural flexibility. No effect of mobility was supported for birds.

**Supplementary Tables**

**Supplementary Table S1.** Set of supported (∆AICc < 2) and best non-supported (∆AICc > 2, between brackets) candidate models for population trends of bird and butterfly species, with their relative fit (log-likelihood) and support (AICc weight and Sum of AICc weights) according to the model selection procedure.

| **Analyses** | **Supported and (best non-supported) models** | | **K** | **Log-likelihood** | **∆AICc** | **AiCc weight** | **Sum AICc weights** |
| --- | --- | --- | --- | --- | --- | --- | --- |
|  | **Main effects** | **Interaction effects** |  |  |  |  |  |
| **Butterflies** | SPI[ibe] + Migrant |  | 4 | 126.2 | 0 | 0.302 | 0.302 |
|  | STI[eur] + SPI[ibe] + Migrant |  | 5 | 126.8 | 1.128 | 0.172 | 0.473 |
|  | SPI[ibe] + SAFI + Migrant |  | 5 | 126.5 | 1.779 | 0.124 | 0.597 |
|  | (STI[eur] + SPI[ibe] + Migrant | STI[eur]×SPI[ibe] | 6 | 127.2 | 2.848 | 0.073 | 0.67) |
| **Birds** | STI[eur] + SPI[ibe] + SAFI + Taxon |  | 15 | 213.9 | 0 | 0.073 | 0.073 |
|  | STI[eur] + SPI[ibe] + SAFI | STI[eur]×SAFI | 6 | 201.9 | 0.063 | 0.071 | 0.144 |
|  | STI[eur] + SPI[ibe] + SAFI + Migrant + Taxon |  | 16 | 215.4 | 0.177 | 0.067 | 0.211 |
|  | STI[eur] + SPI[ibe] + SAFI |  | 5 | 200.7 | 0.241 | 0.065 | 0.275 |
|  | STI[eur] + SPI[ibe] + SAFI + Migrant |  | 6 | 201.7 | 0.551 | 0.055 | 0.331 |
|  | STI[eur] + SPI[ibe] + SAFI + Migrant | STI[eur]×SAFI | 7 | 202.8 | 0.779 | 0.049 | 0.38 |
|  | STI[eur] + SPI[ibe] + SAFI + Taxon | STI[eur]×SAFI | 16 | 214.8 | 1.532 | 0.034 | 0.414 |
|  | (SPI[ibe] + SAFI |  | 4 | 198.7 | 2.032 | 0.026 | 0.441) |

K: number of parameters estimated in the model.

Log-likelihood: relative measure of model fit.

∆AICc: difference in AICc between any candidate model and the best model associated with the smallest AICc.

AICc weight: weight of evidence that the candidate model is the best model.

Predictors: STI[eur] = Species Temperature Index at European scale, SPI[ibe]= Species Precipitation Index at Iberian scale, SAFI = Species Afforestation Index, Mobility (Y/N), Taxon = Family for butterflies and Order for birds (except for the large Order Passeriformes which was split between Infraorder levels).

**Supplementary Table S2.** Results of the AICc-based analysis examining the variations in butterfly and bird species population trends relative to their climatic and land use preferences as well as controlling factors.

| **Effects** | **Predictors** | **Butterflies** | |  |  | **Birds** |  |  |
| --- | --- | --- | --- | --- | --- | --- | --- | --- |
|  |  | **freq** | **w** | **p** |  | **freq** | **w** | **p** |
| **(Intercept)** | | 1 | 1 | 1 |  | 1 | 1 | 1 |
| **Main effects** | |  |  |  |  |  |  |  |
|  | STI[eur] | 0.722 | 0.451 | 0.368 |  | 0.722 | 0.874 | 0.036 |
|  | SPI[ibe] | 0.722 | 0.923 | 0.016 |  | 0.722 | 0.914 | 0.025 |
|  | SAFI | 0.722 | 0.385 | 0.508 |  | 0.722 | 0.899 | 0.032 |
|  | Migrant | 0.5 | 0.944 | 0.005 |  | 0.5 | 0.431 | 0.203 |
|  | Taxon | 0.5 | 0.011 | 0.944 |  | 0.5 | 0.405 | 0.033 |
| **Interaction effects** | |  |  |  |  |  |  |  |
|  | STI[eur]×SPI[ibe] | 0.278 | 0.121 | 0.194 |  | 0.278 | 0.181 | 0.103 |
|  | STI[eur]×SAFI | 0.278 | 0.065 | 0.423 |  | 0.278 | 0.333 | 0.046 |
|  | SPI[ibe]×SAFI | 0.278 | 0.082 | 0.312 |  | 0.278 | 0.195 | 0.096 |

Predictors: STI[eur] = Species Temperature Index at European scale, SPI[ibe]= Species Precipitation Index at Iberian scale, SAFI = Species Afforestation Index, Mobility (Y/N), Taxon = Family for butterflies and Order for birds (except for the large Order Passeriformes which was split between Infraorder levels).

freq: frequency of the different predictors in the list of candidate models.

w: level of importance of the predictor for explaining the data (range: 0-1).

p: probability that by chance w is as high as the estimated value (based on 1000 permutations).
